# The role of elevated lipoprotein(a) in aortic valve disease: a systematic review

**DOI:** 10.3389/fcvm.2025.1610395

**Published:** 2025-10-13

**Authors:** P. Wambua, M. Wahinya, Z. Khan

**Affiliations:** ^1^ Tigoni Level IV Hospital, Kiambu, Kenya; ^2^ Kenyatta University Teaching, Referral & Research Hospital, Nairobi, Kenya; ^3^ Cardiology Department, Bart’s Heart Centre, London, United Kingdom

**Keywords:** lipoprotein(a), aortic stenosis, aortic sclerosis, aortic valve calcification, genetic polymorphisms, cardiovascular disease, targeted therapies

## Abstract

**Background:**

Calcific aortic valve stenosis (CAVS) is the most prevalent valvular heart disease and a growing global health concern. Aortic sclerosis (ASc) and aortic stenosis (AS) represent a continuum of progressive disease characterized by leaflet thickening, inflammation, lipid deposition, and calcification. Lipoprotein(a) [Lp(a)], with its pro-atherogenic, pro-inflammatory, and pro-calcific properties, has emerged as a key contributor to this process. While its role in atherosclerotic cardiovascular disease is well established, the relationship between Lp(a) and CAVS has been demonstrated in several key studies; however, the available evidence remains limited in volume, and important gaps persist in understanding mechanisms, risk stratification, and therapeutic implications.

**Methods:**

A systematic literature search was conducted in PubMed, Cochrane Library, ScienceDirect, Medline, ResearchGate, Embase, and Google Scholar in accordance with PRISMA guidelines. Eligible studies included observational designs (cross-sectional, cohort, case-control) and randomized trials evaluating associations between Lp(a) levels, genetic variants, and CAVS. Study quality was assessed using the Newcastle–Ottawa Scale (NOS).

**Results:**

Eighteen studies met the inclusion criteria, comprising six case-control, six cohort, and six cross-sectional studies with a total of 153,192 participants. No randomized controlled trials were identified. Elevated Lp(a) levels were consistently associated with an increased risk of AS and aortic valve calcification (AVC), with a dose-dependent effect. The risk was highest at levels ≥50 mg/dl, though some evidence supported risk at ≥30 mg/dl. Genetic analyses identified rs10455872 as a significant risk allele, while rs3798220 showed inconsistent associations. Multi-ethnic cohorts highlighted racial variability: Afro-Caribbean individuals had higher baseline Lp(a) levels but lower AVC prevalence than Caucasians.

**Conclusion:**

Lp(a) is an independent risk factor for CAVS, influenced by both concentration and genetic variation. Early screening and emerging Lp(a)-lowering therapies, including antisense oligonucleotides, small interfering RNA, and PCSK9 inhibitors, may help mitigate disease progression. Further randomized trials are needed to determine whether Lp(a) reduction translates into cardiovascular and valvular benefit.

**Systematic Review Registration:**

https://www.crd.york.ac.uk/PROSPERO/view/CRD42024533835, PROSPERO CRD42024533835.

## Introduction

Calcific aortic valve stenosis (CAVS) is the most common valvular heart disease and is projected to impose a substantial health burden in the coming decades (1, 2). Aortic sclerosis (ASc) and aortic stenosis (AS) form a disease continuum, beginning with leaflet thickening and progressing to severe obstruction.

The pathophysiology of CAVS is multifactorial, but lipoprotein(a) [Lp(a)] has emerged as a key driver (4). Lp(a) consists of a low-density lipoprotein–like particle covalently bound to apolipoprotein(a) and carries pro-atherogenic, pro-inflammatory, and pro-thrombotic properties (3, 7, 8). Elevated Lp(a) is an established causal factor in coronary artery disease and myocardial infarction, and increasing evidence implicates it in aortic valve calcification and stenosis (6, 11, 13, 25).

Lp(a) was first described by Berg in 1963 (1), and subsequent advances—including cloning of the LPA gene—clarified its genetic basis (3, 6). Carriers of risk alleles such as rs10455872 and rs3798220 produce smaller apolipoprotein(a) isoforms, leading to higher plasma concentrations and increased risk of calcific valve disease (3, 6, 22). Early studies were hampered by inadequate assays, but modern techniques have confirmed the causal association between Lp(a), oxidized phospholipids, and fibrocalcific remodeling of the aortic valve (13, 25).

Despite these advances, the relationship between Lp(a), race, genetic polymorphisms, and CAVS remains incompletely understood. This systematic review synthesizes the available evidence on the association between Lp(a) levels and the risk of AS and ASc, aiming to clarify its role in disease onset and progression, highlight gaps in knowledge, and inform therapeutic strategies.

## Methods

### Study design

This systematic review was conducted in accordance with PRISMA guidelines (9) and registered with PROSPERO (CRD42024533835).

### Search strategy

**Figure 1:**
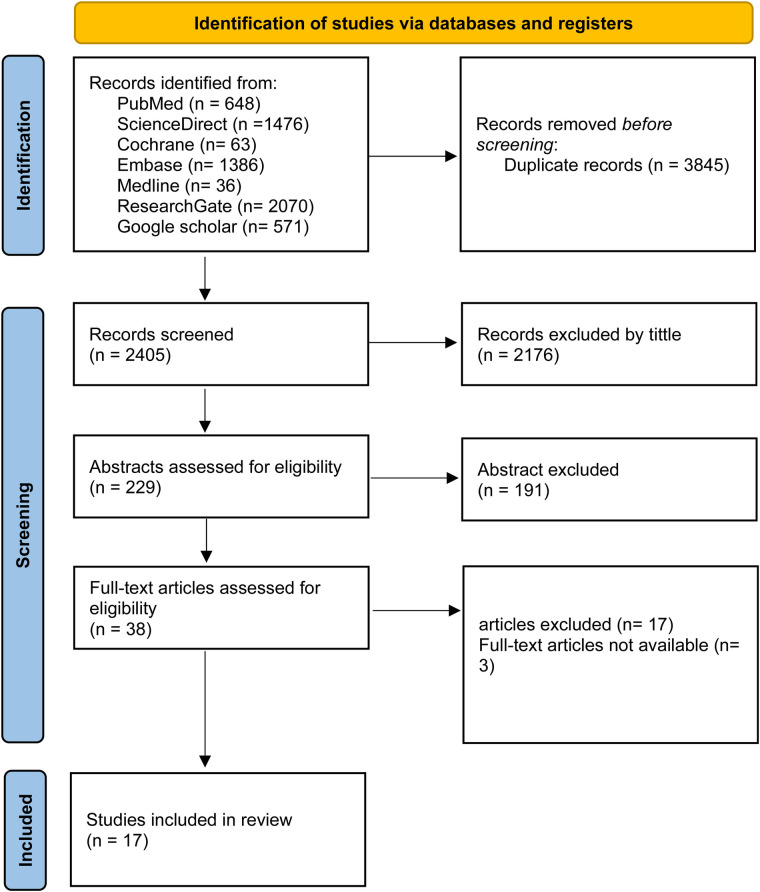
PRISMA flow diagram.

A systematic search was performed across PubMed, Cochrane Library, ScienceDirect, Medline, ResearchGate, Embase, and Google Scholar between June 1st and July 1st, 2024. Keywords and MeSH terms included: lipoprotein(a) OR Lp(a) AND “calcific aortic valve disease,” “aortic valve sclerosis,” “aortic valve stenosis,” “aortic stenosis,” “aortic sclerosis,” and “aortic valve calcification.” The search and screening process is shown in Figure 1.

### Study population and eligibility (inclusion/exclusion)

Eligibility Criteria: The criteria were structured using the PECO framework (10):

○ Population: Adults from the general population.
○ Exposure: Elevated plasma Lp(a) levels or presence of LPA genetic variants.
○ Comparator: Individuals with normal Lp(a) levels or non-risk variants.
○ Outcomes: Development or progression of AS or ASc, or presence of AVC.

Inclusion criteria: observational studies (cohort, case-control, cross-sectional) and RCTs evaluating Lp(a) and calcific aortic valve disease; studies published in English since 2010.

Exclusion criteria: studies of non-calcific valvular disease (e.g., rheumatic), those not reporting Lp(a) in mg/dl, or non-English publications.

### Quality assessment

Methodological quality was assessed using the Newcastle–Ottawa Scale (NOS). Scores of 0–3 indicated high risk, 4–6 moderate, and 7–9 low risk of bias. Two reviewers independently assessed studies, resolving disagreements by consensus with a third reviewer. Sixteen studies were high quality, and two were of moderate quality (Table 1).

**Table 1:** Newcastle-Ottawa quality assessment scale of nonrandomised studies.

Studies	Design	Selection	Comparability	Exposure/Outcome	Overall rating	Quality
Kamstrup et al. (6)	Cohort	**	**	**	7/9	High
Arsenault et al. (12)	Case-control	***	**	*	6/9	Moderate
Zheng et al. (13)	Case-control	****	**	**	8/9	High
Kaltoft et al. (14)	Cohort	***	**	**	7/9	High
Makshood et al. (15)	Cross-sectional	***	**	**	7/10	High
Kaiser et al. (16)	Cohort	****	*	**	7/9	High
Kaiser et al. (17)	Cross-sectional	***	**	***	8/10	High
Liu et al. (18)	Cohort	**	*	***	6/9	Moderate
Burdeynaya et al. (19)	Cross-sectional	**	**	***	7/10	High
Mahabadi et al. (20)	Case-control	***	*	***	7/9	High
Wilkinson et al. (21)	Cross-sectional	****	**	***	9/9	High
Chen et al. (22)	Case-control	****	**	***	9/10	High
Yang et al. (23)	Case-control	****	**	***	9/10	High
Vongpromek et al. (24)	Crosssectional	****	*	***	8/10	High
Capoulade et al. (25)	Cohort	****	*	***	8/9	High
Obisesan et al. (26)	Cohort	***	*	***	7/9	High
Hojo et al. (27)	Case-control	***	**	**	8/9	High
Cao et al. (28)	Crosssectional	***	**	***	8/9	High

*,**,***,****The Newcastle–Ottawa Scale (NOS) is a validated instrument for assessing. The scale is divided into three domains: selection (maximum of 4 points), comparability very good quality: 9–10 points. Good quality: 7–8 points. Satisfactory quality: 5–6 points. Unsatisfactory quality: 0–4 points.

**Figure 2:**
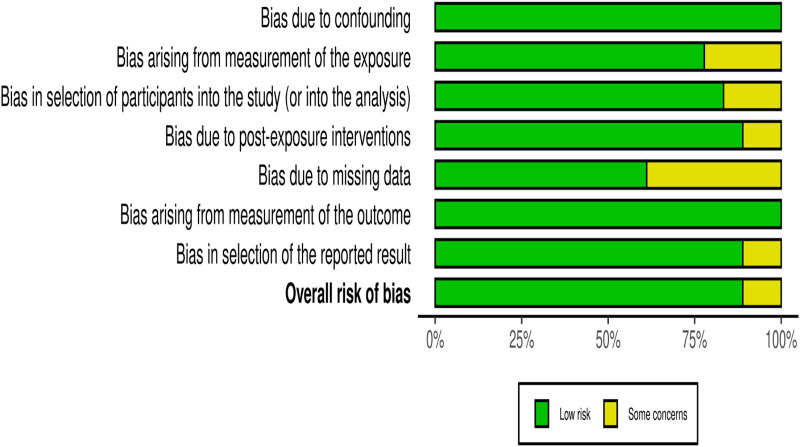
Risk of bias assessment of included studies using the ROBINS.

Figure 2 shows summary plot for risk of bias assessment.

## Results

### Search results and study characteristics

From 6,250 articles screened, 18 studies met inclusion criteria, including six cohorts, six case–controls, and six cross-sectional studies from Europe, the USA, and Asia, with a total of 153,192 participants. Ten studies evaluated Lp(a) and AS, six focused on AVC, and four assessed genetic variants. Participants' mean or median age ranged from 46 to 80 years, with varied sex distribution.

### Lp(a) and aortic valve stenosis

Most studies demonstrated that elevated Lp(a) was associated with higher risk of AS, with thresholds ≥50 mg/dl consistently linked to incident disease (12, 13, 15, 19). A dose–response effect was reported by Kamstrup et al. (6), where risk increased at >20 mg/dl (HR: 1.6, 95% CI 1.1–2.4) and peaked above 90 mg/dl (HR: 2.9, 95% CI 1.8–4.9). In contrast, Mahabadi et al. (20) did not find a significant association.

### Lp(a) and aortic valve calcification

All six studies confirmed an association between elevated Lp(a) and AVC. Higher concentrations correlated with greater calcification severity. Multi-ethnic cohorts highlighted variability: in MESA (28), Afro-Caribbean participants had higher baseline Lp(a) (35 mg/dl) than Hispanics (13 mg/dl), Caucasians (13 mg/dl), and Chinese (12.9 mg/dl). Although Caucasians exhibited lower median levels, they had a higher baseline prevalence of AVC. After adjustment, the association between Lp(a) and AVC persisted in Caucasians but not in other groups. ARIC and Makshood et al. reported similar findings, with associations in Afro-Caribbean and Caucasian populations but not in South Asians (15, 16).

### Association between Lp(a) genetic variants and aortic valve disease

Two Lp(a) genotypes were evaluated. The rs10455872 allele was consistently associated with increased risk of aortic valve stenosis or sclerosis across four studies (6). In contrast, the rs3798220 variant showed no significant association with AVS (6). Chen et al. (22) reported that carriers of two risk alleles (heterozygous, homozygous, or compound heterozygous) had a two-fold higher risk of AVS compared to those with one allele.

Table 2 below shows the characteristics and findings of the included studies.

**Table 2:** Summary of key observational studies evaluating the association.

Studies	Study design	Country	Participants (n)	Mean Age (Years)	Sex (Male %)	Lp(a) levels (mg/dl)	Aim of the study	Findings
Kamstrup et al. (6)	Cohort	Denmark	77,680	58	44	<5 (<22nd percentile) 5–19 (22–66th percentile) 20–64 (67–89th percentile) 65–90 (90–95th percentile) >90 (>95th percentile)	To evaluate the relationship between elevated Lp(a) concentrations and Lp(a) risk genotypes with the likelihood of developing aortic valve stenosis.	A progressive linear increased risk of developing AVS was observed in patients in the upper percentiles of Lp(a) levels (≥22nd percentile) in comparison with the lowest percentile group (<22nd percentile). 22–66th percentile (HR: 1.2, 95% CI: 0.8–1.7) 67–89th percentile (HR: 1.6, 95% CI: 1.1–2.4) 90–95th percentile (HR: 2.0, 95% CI: 1.2–3.4) >95th percentile (HR: 2.9, 95% CI: 1.8–4.9) Patients without rs3798220 allele were at lower risk of AVS compared to patients with rs10455872 allele. Heterozygous (OR: 1.6, 95% CI: 1.2–2.0)Homozygous (OR: 1.5, 95% CI: 0.5–4.8)
Arsenault et al. (12)	Case-control	UK	17,553	60	44	≥50	To determine if Lp(a) levels and its genetic variant rs10455872 G, an allele of Lp(a), are linked to an elevated risk for developing AVS	Lp(a) levels ≥50 mg/dl were independently associated with an increased risk of AVS (HR: 1.98; 95% CI: 1.25–3.09; *p* = 0.002) A significant association was observed between the rs10455872 G allele and an elevated risk of AVS (OR: 1.5; 95% CI: 1.10–2.26)
Zheng et al. (13)	Case-control	UK	17,745	60	45	≥50	To assess for a relationship between Lp(a) levels and incidence of Aortic valve stenosis AVS identified through- Hospitalisation for AVS or death due to AVS	An independent association was found between Lp(a) levels > 50 mg/dl and an increased risk of AVS. (HR: 1.70, 95% CI: 1.33–2.19; *p* < 0.001)
Kaltoft et al. (14)	Cohort	Denmark	12,006	59	43	<10 (0–49th percentile) 10–24 (50–71st percentile) 25–44 (72–80th percentile) 45–69 (81–89th percentile) 70–94 (90–95th percentile) ≥95 (96–100 percentile)	To determine if elevated Lp(a) is causally associated with both Mitral and Aortic valve calcification	Higher levels of Lp(a) were associated with an increases risk for mitral valve calcification: 25–44 mg/dl (OR: 1.04, 95% CI: 0.82–1.3) 45–69 mg/dl (OR: 1.34, 95% CI: 1.09–1.65) 70–94 mg/dl (OR: 1.66, 95% CI: 1.28–2.14) ≥95 mg/dl (OR: 1.79, 95% CI: 1.37–2.25) Higher Lp(a) levels were also associated with increased risk for aortic valve calcification: 25–44 mg/dl (OR: 1.19, 95% CI: 0.99–1.43) 45–69 mg/dl (OR: 1.85, 95% CI: 1.56–2.18) 70–94 mg/dl (OR: 2.23, 95% CI: 1.81–2.75) ≥95 mg/dl (OR: 3.01,95% CI: 2.45–3.68) Patients with Lp(a) genetic variants that are associated with higher levels of Lp(a) also showed increased risk for aortic valve calcification: rs10455872 (OR: 1.82, 95% CI: 1.64–2.13) rs3798220 (OR: 1.52, 95% CI: 1.13–2.04)
Makshood et al. (15)	Cross-sectional	USA	5,366	59	43	>30 vs. >50	To determine an association between Lp(a) levels and AVC among South Asians compared to other races/ethnic groups. The assessment was done using a cardiac CT scan.	No statistically significant association was observed between the levels of Lp(a) and the development and severity of AVC among the South Asian population. However, continuously elevated Lp(a) levels showed an increased risk for AVC among the Afro-Caribbean individuals (OR: 0.14, 95% CI: 0.06–0.22; *p* 0.0009) and Caucasian individuals (OR: 0.13, 95% CI: 0.07–0.20; *p* < 0.0001)
Kaiser et al. (16)	Cohort	Netherlands	922	66	48	—	To assess the relationship between Lp(a) levels and the incidence or progression of aortic valve calcium (AVC) AV was assessed at baseline and after a median follow-up of 14 years using non-enhanced cardiac computed tomography.	Each ≥50 mg/dl increase in Lp(a) concentration was independently associated with the development of new-onset AVC (OR: 1.3, 95% CI: 1.02–1.65). However, Lp(a) levels were not associated with the progression of AVC. Lp(a) plasma levels ≥50 mg/dl were associated with AVC at baseline (OR: 1.4, 95% CI: 1.15–1.79)
Kaiser et al. (17)	Cross-sectional	Netherlands	3,271	70 (Rotterdam Study cohort) 46 (Amsterdam University Medical Centres)	48	<12.5 (<50th percentile) 12.5–47.7 (50–79th percentile) 47.7–88.7 (80–94th percentile) >88.7 (≥95th Percentile)	To determine the association between Lp(a) and AVC in 2 large cohorts: the Rotterdam Study cohort & the Amsterdam University Medical. Centres (UMC) outpatient clinic Assessed by non-enhanced Cardiac CT SCAN	Elevated Lp(a) concentrations per 50 mg/dl increase were independently associated with the AVC in the Rotterdam Study cohort. (OR: 1.54; 95% CI: 1.36–1.75) Amsterdam UMC cohort (OR: 2.02;95% CI: 1.19–3.44)
Liu et al. (18)	Cohort	China	652	62	58	>38	Association between Lp(a) levels and severity of aortic stenosis Baseline assessment done by ECHO Follow-up assessment: AV replacement or death from AVS.	Patients with higher Lp(a) levels at baseline had a significantly higher risk of severe AS (OR: 1.78; 95% CI: 1.18–2.66, *P* 0.006) On follow-up (mean 3.16 ± 2.74 yrs), Lp(a) was not associated with AVR or death from AVS.
Burdeynaya et al. (19)	Cross-sectional	Russia	250	69	42	>30	“To determine the role of Lp(a) and its autoantibodies in CAVS in patients with and without coronary heart disease.”	Increasing Lp(a) levels ≥30 mg/dl were associated with CAVS (OR: 3.7, 95% CI: 1.8–7.3; *p* < 0.001) Autoantibodies (IgM) to oxidised Lp(a) were associated with CAVS irrespective of Lp(a) levels.
Mahabadi et al. (20)	Case-control	Germany	968	80	52	—	To compare the Lp(a) levels of patients with and without AVS	No difference in Lp(a) level was observed in patients with and without AVS.
Wilkinson et al. (21)	Case-control	US	4,079	75	53	≥30	Prevalence of Lp(a) measurement and degree of elevation among patients with Aortic stenosis based on echo assessment.	66% of patients with AS had Lp(a) levels <30 mg/dl, 14% had Lp(a) levels between 30 and 60 mg/dl and, 20% had Lp(a) levels >60 mg/dl
Chen et al. (22)	Case-control	USA	3,469	74	56	—	‘To determine the association of Lp(a) variants (rs10455872 and rs3798220) with Aortic stenosis’	Lp(a) variants were associated with an increased risk of developing aortic stenosis: Per risk allele: rs10455872 (OR: 1.34, 95% CI: 1.23–1.47) rs3798220 (OR: 1.31, 95% CI: 1.09–1.58) Two risk alleles: Homozygous rs10455872 (OR: 2.05, 95% CI: 1.37–3.07) Homozygous rs3798220 (OR: 3.74, 95% CI: 1.03–1.36) Compound heterozygotes (OR: 2.0, 95% CI: 1.17–3.44)
Yang et al. (23)		China	1,260	Young aged (30–59) 55 Middle-age (60–74) 68 Elderly (75–93) 79	42	—	To evaluate the correlation between serum Lp(a) levels and incidence of ACS	Aging, LDL-C and Lp(a) were all demonstrated to be risk factors for AVS (*β*= 0.04, 0.222, 0.011 respectively, all *P* < 0.01) Serum Lp(a) levels were shown to be higher among patients with AVS compared with the control group (all *p* < 0.05)
Vongpromek et al. (24)	Cross-sectional	Netherlands	129	51	63	—	To look for an association between Lp(a) levels and AVC in asymptomatic statin-treated patients with heterozygous familial hypercholesterolemia. Assessment using non-non-enhanced CT scan	Increasing Lp(a) levels (every 10 mg/dl increment of Lp(a) concentration) was associated with AVC risk (OR: 1.11; 95% CI: 1.01–1.2; *P* = 0.03)
Capoulade et al. (25)	Cohort	Canada	220	58	60	58.5	To determine whether Lp(a) and oxidised phospholipids are associated with AS progression and AS-related events	Patients with Lp(a) levels >58.5 mg/dl had significant progression of aortic stenosis compared to those with Lp(a) levels ≤58.5 mg/dl.
Obisesan et al. (26)	Cohort	US	2,083	59	38	>50	To evaluate the association between Lp(a) and subclinical vascular and valvular calcification from the ARIC study with evaluation performed by using cardiac CT scans.	Lp(a) levels >50 mg/dl significantly increased the odds of aortic valve calcification (OR: 1.82; 95% CI: 1.34–2.47) after adjusting for cardiovascular risk factors and the use of lipid lowering therapy.
Hojo et al. (27)	Case-control	Japan	861	73	80	34	To evaluate the association between Lp(a) and aortic and mitral valve stenosis in patients with PAD based on echo evaluation.	Patients with AS had higher Lp(a) levels compared to patients without AS [34.0 (16.7–50.0) vs. 20.0 (11.0–35.0) mg/dl, *P* = 0.002],
Cao et al. (28)	Cross-sectional	USA	4,678	Median 61–62	45	≥30 vs. ≥50	To determine the Lp(a) cut-off values that identify risk for calcific aortic valve disease (CAVD) across multiple ethnicities based on CT scan assessment of the AV calcium score.	Lp(a) cut-off values of 30 mg/dl were associated with CAVD in the Caucasian population (RR 1.56, 95% CI: 1.24–1.96). In the Afro-Caribbean population, cut-off values of 30 mg/dl had a borderline association with CAVD. There was no significant association between the Hispanic and Chinese population. Cut-off values of 50 mg/dl showed a significant association with CAVD in the Caucasian population (RR 1.72, 95% CI: 1.36–2.17), but no significant association was found with other races.

## Discussion

This systematic review confirms a robust, dose-dependent association between elevated Lp(a) and CAVD, despite heterogeneity across study designs. Concentrations ≥50 mg/dl were reliably associated with disease risk (12, 13, 15, 19), while thresholds around ≥30 mg/dl yielded mixed findings (12, 15, 20). Elevated Lp(a) has also been implicated in disease progression, accelerating hemodynamic deterioration and adverse outcomes (13, 25), although Kaiser et al. (16) observed associations only with incident, not progressive, AVC.

Genetic determinants provide strong causal evidence. Variants such as rs10455872 consistently predict elevated Lp(a) and higher risk of CAVD (3, 6, 22). Carriage of multiple risk alleles more than doubled AS risk, with stronger effects in younger and male populations (12, 22). Instrumental variable analyses suggest a relative genetic risk of 1.6 for AS with a tenfold increase in Lp(a) (5, 6). These findings align with the biological mechanism whereby fewer kringle IV type 2 (KIV-2) repeats produce smaller apo(a) isoforms that are synthesized at higher rates, leading to elevated plasma concentrations.

Ethnic variability was also evident. Afro-Caribbean and Caucasian individuals demonstrated the strongest associations (15, 28), whereas South Asian, Hispanic, and Chinese populations showed weaker or inconsistent links (15, 28). Interestingly, despite lower median levels in Caucasians compared to Afro-Caribbeans, subclinical CAVD was more prevalent in the former (15), suggesting complex gene–environment interactions.

Mechanistically, Lp(a) is increasingly recognized as a multifactorial driver of CAVD. Circulating Lp(a) carries oxidized phospholipids (OxPLs), which promote endothelial activation, inflammatory cell infiltration, and osteogenic signaling (e.g., bone morphogenetic proteins), thereby accelerating valve fibrosis and calcification in a manner paralleling atherosclerosis (26, 27). Oxidized Lp(a) also impairs fibrinolysis and potentiates thrombosis, further contributing to valvular injury. A conceptual framework can thus be summarized:

Geneticvariants→Smallerapo(a)isoforms→IncreasedhepaticsynthesisofLp(a)→Elevatedplasmalevels→CarriageofOxPLs→Inflammationandfibrosis→Valvularcalcification→CAVDprogression.

Therapeutically, conventional lipid-lowering therapies have limited impact on Lp(a). Statins are ineffective and may modestly increase levels (29, 30), whereas PCSK9 inhibitors provide modest reductions (∼20%–25%) and have demonstrated cardiovascular benefit in outcomes trials, partly attributable to Lp(a) lowering (31, 32). More potent agents are in development: siRNAs (Olpasiran) reduce Lp(a) by up to 90% (33) and are under evaluation in the Phase 3 OCEAN[a]-Outcomes trial (NCT05581303); ASOs (Pelacarsen) reduce Lp(a) by ∼80% (34), with the large HORIZON trial underway. ANGPTL3 inhibitors also show potential in lowering Lp(a) alongside other lipids (35). These developments highlight a paradigm shift toward precision therapeutics for genetically mediated risk factors such as Lp(a).

Overall, the evidence indicates that Lp(a) is not only a biomarker but also a causal mediator of CAVD, supported by consistent epidemiological, genetic, and mechanistic data. These insights reinforce the rationale for incorporating Lp(a) into risk stratification models and prioritizing Lp(a)-specific therapies to prevent both cardiovascular and valvular events.

### Future directions

Future research should focus on integrating Lp(a) measurement into routine cardiovascular and valvular risk assessment, especially for individuals with a family history of premature atherosclerotic disease or CAVD. Large-scale registries and multi-ethnic cohorts are required to clarify ancestry-specific risks, as evidence suggests important variability across populations. Mechanistic studies should further elucidate the roles of oxidized phospholipids, inflammation, and valve interstitial cell activation in disease progression, which may uncover new therapeutic targets. Most importantly, the outcomes of ongoing Phase 3 trials (HORIZON, OCEAN[a]-Outcomes) will determine whether targeted Lp(a) lowering can alter the natural history of CAVD. If successful, these therapies could establish a new standard of care, shifting management from late-stage intervention to early, precision-based prevention.

### Limitations

This review has several limitations. First, it included only observational studies, and no randomized controlled trials (RCTs) are yet available to establish causality between elevated Lp(a) and CAVD. Second, the included studies were conducted predominantly in high-income countries (Europe, the United States, China, and Japan), with limited data from developing regions and Sub-Saharan Africa, restricting global generalizability. Third, although the overall risk of bias was low, there was significant heterogeneity in study design, population characteristics, and Lp(a) thresholds, which may influence interpretation.

## Conclusion

In summary, elevated Lp(a) is consistently associated with an increased risk of atherosclerotic cardiovascular disease, aortic valve sclerosis, and stenosis, supported by genetic and mechanistic evidence. While RCTs confirming that lowering Lp(a) reduces CAVD risk are lacking, emerging therapies such as siRNAs and antisense oligonucleotides offer great promise. Large, multi-ethnic RCTs are urgently needed to determine whether targeted Lp(a) reduction can modify the natural history of CAVD and should specifically include underrepresented populations such as Sub-Saharan Africa. Establishing effective Lp(a)-directed interventions could transform management paradigms, moving from symptomatic treatment of advanced valve disease to early, precision-based prevention.

## Data Availability

The original contributions presented in the study are included in the article/Supplementary Material, further inquiries can be directed to the corresponding author.
